# The role of Toll-like receptors and neuroinflammation in Parkinson’s disease

**DOI:** 10.1186/s12974-022-02496-w

**Published:** 2022-06-06

**Authors:** Arash Heidari, Niloufar Yazdanpanah, Nima Rezaei

**Affiliations:** 1 Students’ Scientific Research Center, Tehran University of Medical Sciences, Tehran, Iran; 2 Research Center for Immunodeficiencies, Children’s Medical Center Hospital, Tehran University of Medical Sciences, Dr. Qarib St, Keshavarz Blvd, Tehran, 14194 Iran; 3 Network of Immunity in Infection, Malignancy and Autoimmunity (NIIMA), Universal Scientific Education and Research Network (USERN), Tehran, Iran; 4 Department of Immunology, School of Medicine, Tehran University of Medical Sciences, Tehran, Iran

**Keywords:** Parkinson’s disease, Neuroinflammation, Toll-like receptor, Neurodegeneration

## Abstract

**Background:**

Parkinson’s disease (PD) is the second most prevalent neurodegenerative disorder, characterized by motor and non-motor symptoms, significantly affecting patients’ life. Pathologically, PD is associated with the extensive degeneration of dopaminergic neurons in various regions of the central nervous system (CNS), specifically the substantia nigra. This neuronal loss is accompanied by the aggregation of misfolded protein, named α-synuclein.

**Main text:**

Recent studies detected several clues of neuroinflammation in PD samples using postmortem human PD brains and various PD animal models. Some evidence of neuroinflammation in PD patients included higher levels of proinflammatory cytokines in serum and cerebrospinal fluid (CSF), presence of activated microglia in various brain regions such as substantia nigra, infiltration of peripheral inflammatory cells in affected brain regions, and altered function of cellular immunity like monocytes phagocytosis defects. On the other side, Toll-like receptors (TLRs) are innate immune receptors primarily located on microglia, as well as other immune and non-immune cells, expressing pivotal roles in recognizing exogenous and endogenous stimuli and triggering inflammatory responses. Most studies indicated an increased expression of TLRs in the brain and peripheral blood cells of PD samples. Besides, this upregulation was associated with excessive neuroinflammation followed by neurodegeneration in affected regions. Therefore, evidence proposed that TLR-mediated neuroinflammation might lead to a dopaminergic neural loss in PD patients. In this regard, TLR2, TLR4, and TLR9 have the most prominent roles.

**Conclusion:**

Although the presence of inflammation in acute phases of PD might have protective effects concerning the clearance of α-synuclein and delaying the disease advancement, the chronic activation of TLRs and neuroinflammation might lead to neurodegeneration, resulting in the disease progression. Therefore, this study aimed to review additional evidence of the contribution of TLRs and neuroinflammation to PD pathogenesis, with the hope that TLRs could serve as novel disease-modifying therapeutic targets in PD patients in the future.

## Introduction

Parkinson’s disease (PD) is a progressive neurodegenerative disorder characterized by a wide variety of motor and non-motor symptoms [1]. The classical triad of motor symptoms in PD is bradykinesia, rest tremor, and rigidity. These cardinal symptoms might be preceded by different non-motor features, including sleep disorder, autonomic dysfunction, hyposmia, and depression [2]. Moreover, the disease could be accompanied by cognitive dysfunction, dementia, and poor balance in advanced stages [3]. The PD prevalence increases with age. In this regard, its prevalence rate is estimated to escalate from 1% in adults over 60 years of age to 3–4% in adults older than 85 [4, 5]. Therefore, considering the aging of the global population, the PD economic and societal burden is expected to increase in the following decades rapidly [4].

Pathologically, PD manifestations are associated with the extensive degradation of dopaminergic (DA) neurons in distinctive brain regions, spinal cord, and peripheral nerves [2]. These degenerative changes have been mostly observed in the midbrain’s substantia nigra pars compacta (SNc) [2]. It has been recently recognized that Lewy bodies, containing the aggregation of misfolded proteins named α-synuclein, accompany the neuronal loss in affected regions [6]. Scientists have historically made several hypotheses regarding the degeneration mechanisms of dopaminergic neurons in PD, among which oxidative stress, excitotoxic mechanisms, and environmental factors have a probable role [2]. However, the lack of disease-modifying treatments based on these mechanisms necessitates further exploration of the underlying causes of this disease. The evidence of neuroinflammation in PD in recent years has attracted much attention. This evidence includes increased proinflammatory cytokines in CSF and blood, detection of activated microglia and macrophages in SNc, infiltration of T lymphocytes in affected brain areas, and altered activity of T and B lymphocytes in PD patients [7, 8]. It has been recently found that Toll-like receptors (TLRs) might serve as significant immune receptors in PD, through which neuroinflammation could be triggered.

TLRs are innate immune system receptors, presenting in various human cells and tissues, including immune, epithelial, neural, and glial cells [9]. They are a member of the pattern recognition receptor (PRR) family and play different roles in recognizing pathogen-associated molecular patterns (PAMPs) expressed by microbial invaders and damage-associated molecular patterns (DAMPs), which are endogenous substances released during tissue damage [9]. TLRs, expressing primarily on microglia, can be stimulated during the course of PD through several molecules such as α-synuclein and heat shock proteins (HSPs) like HSP90 [10], HSP70 [11], and HSP60 [12] presented from affected neurons [9]. Their stimulation may activate several downstream pathways involving myeloid differentiation primary response 88 (MyD88) and nuclear factor kappa-light-chain-enhancer of activated B cells (NF-kB), contributing to the production of several proinflammatory cytokines and oxidative stress molecules, leading to neuroinflammation [13]. In this way, TLRs may play pivotal roles in the neural loss in PD pathogenesis. Therefore, this review aims to explore the evidence of the involvement of TLRs in the pathophysiology of PD and their potential therapeutic efficacy.

## An overview of TLRs

### TLRs' structure and functions

TLRs are a group of pattern recognition receptors (PRRs) with central roles in initiating immune responses against different internal and external stimuli. The TLRs’ immune functions were firstly described in *Drosophila* mutants in TLR genes, showing high susceptibility to fungal infections [14]. Up to now, ten members of the TLR family have been recognized in the human species, compared with at least 13 members in rodents [15]. Accordingly, each species has a various number of TLR genes, as shown in *Caenorhabditis elegance* and *Drosophila* to have one and nine TLR genes, respectively [16]. All TLRs are class I transmembrane receptors, consisting of extracellular, transmembrane, and intracellular domains. The extracellular N-terminal domain of TLRs consists of numerous leucine-rich repeats (LRR) and cysteine-rich domains [15–17]. The intracellular section contains an evolutionary-conserved carboxy-terminal domain, denominated Toll/interleukin-1 receptor (TIR) domain, interacting with the TIR or death domain (DD) of adaptor molecules [17]. These receptors are expressed in a variety of immune and non-immune cells, including macrophages, lymphocytes, dendritic cells (DCs), granulocytes, hematopoietic stem cells (HSCs), epithelial cells, neuronal cells, and endothelial cells [18–20]. They usually function as homodimers, with the exception of TLR2, which forms heterodimers with TLR1, or TLR6, each showing a different ligand specificity [21]. In humans, four TLRs, including TLR3, TLR7, TLR8, and TLR9, are located within the endosomal membrane, while the other six TLRs reside in the cells' plasma membrane [20]. Generally, plasma membrane-resided TLRs (TLR 1, 2, 4, 5, 6, and 10) recognize bacterial-related lipid, protein, and lipoprotein compartments, including bacterial flagellin, lipoteichoic acid (LTA), lipopolysaccharide (LPS), di-acylated proteins, and tri-acylated lipoproteins [22]. Each TLR type is sensitive to a specific type of mentioned bacterial compartments; for example, TLR4 can specifically recognize Gram-negative bacterial LPS [22]. Moreover, for some TLRs, ligand binding is facilitated by coreceptors and accessory molecules, including the cluster of differentiation (CD) 12, CD14, and myeloid differentiation factor 2 (MD-2) [15, 23]. CD14 exists in two major forms, namely membrane CD14 (mCD14) and soluble CD14 (sCD14) [24]. In this regard, mCD14, expressed mainly on monocytes and macrophages, is more substantially involved in attaching to LPS. It should be noted that LPS is presented to mCD14 and TLR4 by Lipopolysaccharide binding protein (LBP) [25]. However, it has been shown that both sCD14 and LBP are crucial for LPS-induced TLR activation [26, 27]. Alternatively, endosome-associated TLRs (TLR 3, 7, 8, and 9) are specific to both self-derived and pathogen-related nucleic acids, like viral double- or single-stranded RNAs, and bacterial- or viral-related cytosine phosphate guanine (CpG) motif of DNA [28, 29].

### TLRs' general signaling pathways

Activation of TLRs by binding to their specific ligands initiates downstream signaling cascades, ultimately leading to various cell responses, including the production of proinflammatory cytokines and interferons. Following engagement to their ligands, TLRs experience a conformational change and recruit intracellular adaptive molecules of myeloid differentiation primary response 88 (MyD88), or TIR-domain-containing adaptor-inducing interferon-β (TRIF). In this context, TRAM (TRIF-related adapter molecule) and TIRAP (TIR domain-containing adapter protein) could facilitate the recruitment of TRIF and MyD88 [30]. Depending on the recruited adaptor molecule, different downstream signaling pathways follow. In this regard, most TLRs utilize MyD88 as an adaptive molecule, except for TLR3, which uses the TRIF-dependent pathway. TLR4 can utilize both MyD88 and TRIF pathways [31]. Through the MyD88-dependent pathway, activated TLR recruits MyD88, followed by three members of the serine-threonine kinase interleukin-1 receptor-associated kinase (IRAK) family, including IRAK1, IRAK2, and IRAK4 [32], forming a complex called "Myddosome" [20]. Myddosome then recruits and stimulates the E3 ubiquitin ligase tumor necrosis factor (TNF) receptor-associated factor 6 (TRAF6) by K63-related autoubiquitylation. Subsequently, TRAF6 recruits and activates the transformation growth factor beta-activated kinase 1 (TAK1) and IkB kinase (IKK) by ubiquitination of its IKKγ subunit. TAK1 then activates mitogen-activated protein kinases 3/6 (MKK3/6), MKK4/7, and the IKKb subunit of IKK. After that, MKK3/6, MKK4/7, and IKK activate P38 mitogen-activated protein kinase (MAPK), Jun N-terminal kinases (JNKs), and P65 NF-kB, respectively, leading ultimately to the transfer of these transcription factors to the nucleus and transcription and synthesis of proinflammatory cytokines such as IL-1, IL-6, and TNF-α [15, 20].

Alternatively, the activation of the endosome-resided TLR3 or cell surface TL4 recruits TRIF adaptor protein, which next attaches and activates TNF receptor-associated factor 3 (TRAF3) by using polyubiquitination. Activated TRAF3 recruits and stimulates IKKε and TANK-binding kinase 1 (TBK1). TBK1 then activates IFN regulatory factor 3 (IRF3), ultimately leading to type-I interferon production. It should be mentioned that TRIF also causes the MyD88-independent activation of TRAF6, leading to cytokine synthesis. Moreover, TRAF6 activated by the TLR4-MyD88 pathway can cause cellular inhibitor of apoptosis proteins (CIAPs) modification through K63-linked polyubiquitin. Modified CIAPs then contribute to proteasomal degeneration of TRAF3. The other endosome-resided TLRs, including TLR7, TLR8, and TLR9, signal through MyD88-dependent pathway and can thereby lead to cytokine production; besides, they can activate IFN regulatory factor 3 (IRF3), leading to interferon production [15, 20].

**Fig. 1:**
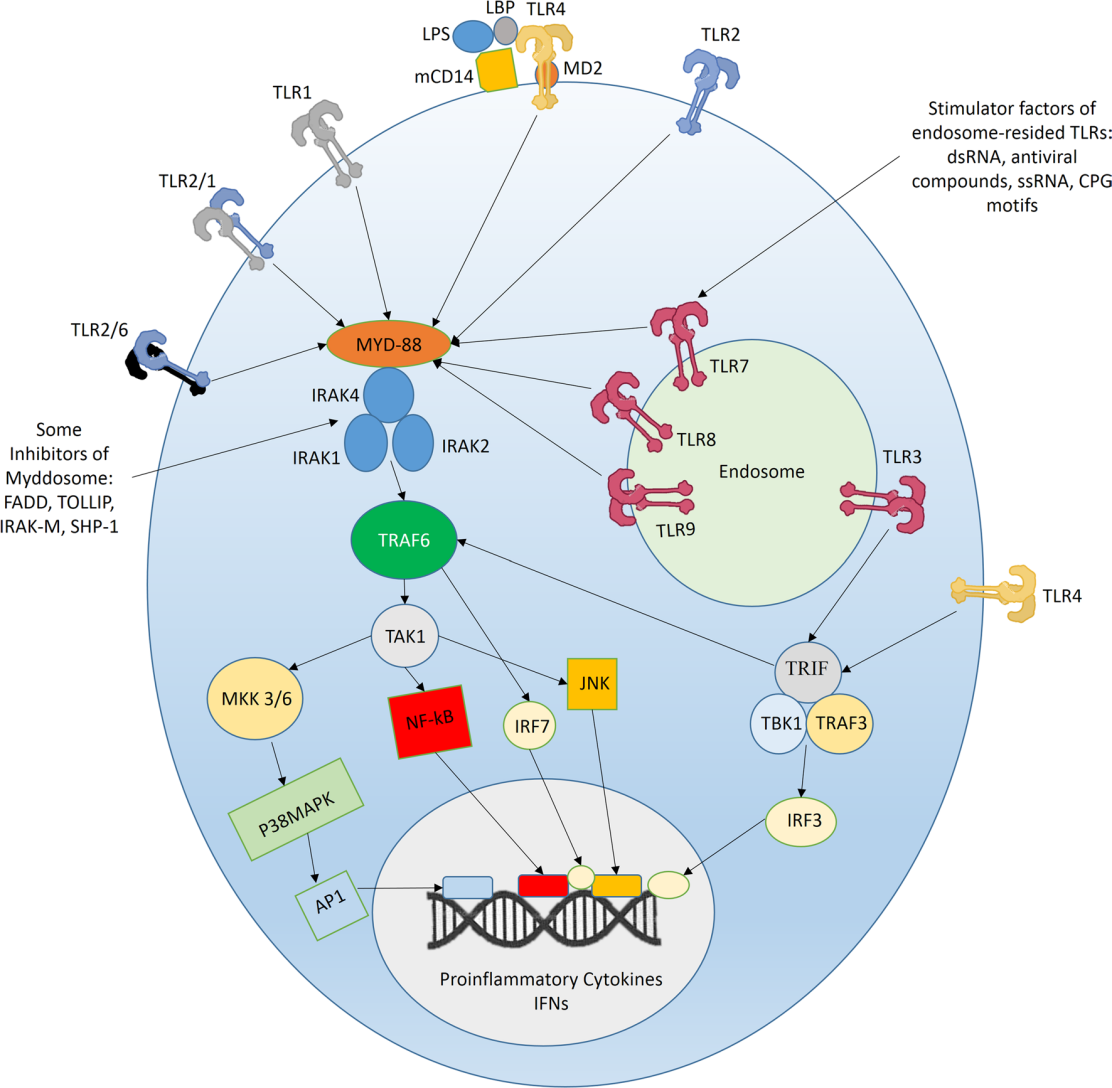
By attaching to their ligands and experiencing conformational changes, all TLRs except for TLR3 recruit the MyD88 adaptive molecule. MyD88 then activates and recruits IRAK1, IRAK2, and IRAK4, forming a complex named Myddosome. Myddosome then recruits and activates TRAF6, followed by activation of TAK1. The activation of TAK1 resulted in the activation and transfer of different transcription factors, including NF-kB, JNK, and AP1, leading to proinflammatory cytokine production. Moreover, TRAF6 can activate IRF7 leading to interferons production. On the other side, TLR3 utilizes the TRIF adaptive molecule, which by activating TRAF3 and TBK leads to activation and transfer of IRF3 to the nucleus and interferons production. Moreover, TRIF can activate TRAF6 and result in MyD88-independent proinflammatory cytokine production. Furthermore, TLR4 can use both MyD88 and TRIF adaptor proteins. It should be mentioned that some TLRs like TLR4 can use coreceptors like CD14 and MD2 for attaching more efficiently to their ligands. Myddosome can be inhibited by several factors, including FADD, TOLLIP, IRAK-M, and SHIP-1. For instance, FADD can antagonize MyD88 or IRAK molecules and their interaction, IRAK-M can inhibit IRAK1 molecule, and TOLLIP can inhibit IRAK phosphorylation and kinase activity [33, 34]. Other examples of TLR regulators are SH2-containing inositol-5′-phosphatase (SHIP) 1 and SHIP2, which contribute to dephosphorylation and inactivation of IRAK1 and TBK1. *TLR* toll-like receptor, *LPS* lipopolysaccharide, *CD14* cluster of differentiation 14, *MD2* Myeloid Differentiation factor 2, *MyD88* Myeloid differentiation primary response 88, *IRAK4* Interleukin-1 receptor-associated kinase, *TRAF6* TNF (tumor necrosis factor) receptor-associated factor 6, *TAK1* transforming growth factor b-activated kinase 1, *MKK* mitogen-activated protein kinase kinase, *JNK* Jun N-terminal Kinase, *IRF* Interferon regulatory factor, *P38MAPK* P38 mitogen-activated protein kinase, *AP1* Activating Protein-1, *NF-kB* nuclear factor kappa-light-chain-enhancer of activated B cells, *TRIF* TIR-domain-containing adaptor-inducing interferon-β, *LBP* Lipopolysaccharide binding protein, *TIR* Toll/interleukin-1 receptor, *TANK* binding kinase 1, *FADD* Fas-associated protein with death domain, *TOLLIP* toll interacting protein, *SHIP* SH2-containing inositol-5′-phosphatase, *dsRNA* double-stranded RNA, *ssRNA* single-stranded RNA

Besides, some inhibitor regulators have been found to negatively affect TLR signaling. For example, Fas-associated protein with death domain (FADD), Toll interacting protein (TOLLIP), and IRAK-M can prevent Myddosome from being activated. In this regard, FADD can antagonize MyD88 or IRAK molecules and their interaction, IRAK-M can inhibit IRAK1 molecule, and TOLLIP can inhibit IRAK phosphorylation and kinase activity [33, 34]. Other examples of TLR regulators are SH2-containing inositol-5′-phosphatase (SHIP) 1 and SHIP2, which contribute to dephosphorylation and inactivation of IRAK1 and TBK1. Furthermore, tumor necrosis factor α-induced protein 3 (TNFAIP3 or A20) can inhibit TRAF6 and IKK activation [15, 35]. The mentioned TLR regulator proteins are named as some bold examples of TLR signaling inhibitory adaptor molecules, which are naturally expressed by genes located in the human genome. These molecules integrate with the TLR signaling pathway to regulate inflammatory responses to different stimuli (Fig. 1).

### TLR roles in inflammation and neuroinflammation

Recent studies have questioned the common belief regarding considering the central nervous system (CNS) as a complete immune-privileged organ. In the CNS, there are various resident cell types such as microglia, astrocytes, and oligodendrocytes performing roles as innate immune cells [36]. The activation of these cells under several conditions can contribute to the secretion of inflammatory cytokines and reactive oxygen species (ROS), leading to neuroinflammation, which might disrupt the blood–brain barrier (BBB) and thereby allow and chemotactically attract several peripheral immune cells such as monocytes, macrophages, neutrophils, and T cells to join the CNS, aggravating the inflammation [37–39]. This inflammation might be triggered due to different CNS infections or sterile aggregation of toxic metabolites and proteins, as seen in neurodegenerative disorders. Though the presence of neuroinflammation is necessary for the clearance of pathological invaders or toxic metabolites accumulated in the CNS, the uncontrolled stimulation of this inflammation might propagate the condition like what can be seen during the courses of meningitis, PD, or Alzheimer’s disease [38]. TLRs expressed by the CNS-located immune or neural cells were shown to have substantial roles in inducing and maintaining neuroinflammation. Therefore, they could serve as double-edged swords in the pathogenesis of many CNS-related diseases [38].

Bacterial meningitis, viral infections, or sterile inflammatory disorders like Alzheimer's disease or cerebral ischemia can be named as examples of the involvement of TLRs in neuroinflammation. Bacterial meningitis can be caused by Gram-positive (G +) bacteria like *Streptococcus pneumonia* (*S. pneumonia*) and *S. agalactiae*, or Gram-negative (G−) bacteria like *Neisseria meningitides* (*N. meningitides*) and *Escherichia coli* (*E. coli*) [40]. The lipoteichoic acid (LTA), peptidoglycan (PGN), different lipoproteins or proteins of G + bacteria, and LPS of G- bacteria can serve as PAMPs to TLRs residing in the CNS, thereby can lead to excessive transcription of inflammatory cytokine-related genes that result in neuroinflammation followed by meningitis [41]. For example, the porin B protein of *N. meningitides* can cause the formation and stimulation of the TLR2/TLR2 homodimers on the CNS cells, thereby initiating downstream pathways resulting in the activation of NF-kB transcription factor and secretion of proinflammatory cytokines, leading to meningitis [42]. Alternatively, the LPS of G- bacteria and the bacterial DNA can trigger the inflammatory pathway by stimulating TLR4 and TLR9, respectively [38]. On the other hand, several viral infections can contribute to CNS inflammation. In this regard, double-stranded RNA (dsRNA), single-stranded RNA (ssRNA), and non-methylated CpG motifs of invader viruses can stimulate TLR3, TLR7, TLR8, and TLR9 as PAMPs. The stimulation of TLR3 causes the activation of the TRIF-dependent downstream pathway, while TLR7, TLR8, and TLR9 act via the MyD88-dependent pathway. Both of these pathways then contribute to the secretion of inflammatory cytokines and type-I interferon, leading to neuroinflammation [38, 43].

Unlike the mentioned infectious diseases, in plenty of sterile CNS conditions, including neurodegenerative disorders, the presence of toxic metabolites and proteins as intrinsic substances can trigger TLRs and initiate neuroinflammation. For instance, during the course of Alzheimer's disease, the most prevalent progressive neurodegenerative disorder characterized by memory loss and cognitive dysfunction, aggregation of extracellular plaques of amyloid-beta (Aβ) and the neurofibrillary tangles that consist of tau protein have the potential to exert neurotoxic effects and contribute to the pathogenesis of the disease [44]. These accumulated proteins can be recognized as DAMPs and thereby trigger TLRs presenting on different CNS-resided cells, which is necessary for the clearance of these accumulated proteins. However, the overactivation of TLR-dependent pathways by these proteins can detrimentally generate neuroinflammation, which is pathologic. As evidence of TLR involvement during AD, upregulation of *TLR1*, 2, 3, 4, 5, and 6 genes in the temporal cortex of AD patients can be mentioned [45]. It has been found that TLR2 is the primary receptor for the Aβ42 protein, responsible for neuroinflammation during AD, as its deficiency leads to subsided inflammation in this disease [45]. Cerebrovascular accidents might serve as another example of TLR-dependent inflammation in a sterile condition. As cerebral ischemia occurs, the lack of sufficient oxygen supply to the brain cells leads to the release of different intracellular factors, including hyaluronic acid, high-mobility group box 1, heat shock proteins like HSP70, and mRNAs. These released factors are recognized as DAMPs by TLRs residing on microglia, astrocytes, and other CNS cells, leading to proinflammatory cytokine secretion. For instance, heat shock protein 70 (HSP70) was shown to activate TLR2 and TLR4 downstream pathways [38]. The activation of the microglia-resided TLRs can also upregulate specific chemokines, including MCP-1 or CCL3, which are effective in the chemoattraction of peripheral immune cells to the injured area of the CNS [46]. Although some secreted factors such as neurotrophins caused by the activation of TLRs during cerebral ischemia were shown to have neuroprotective effects in animal studies, clinical trials on the efficacy of these neurotrophic factors in humans have failed so far [47, 48].

## Evidence of neuroinflammation in PD

In 1988, McGeer et al*.* were the first to describe evidence of neuroinflammation in PD. They reported the presence of activated microglia and infiltrating lymphocytes in the substantia nigra of PD patients [7]. Although McGeer's way had not been continued for nearly 20 years, in 2008, Brochard et al*.* demonstrated and quantified the evidence of infiltrated T lymphocytes (CD8^+^ and CD4^+^) both in postmortem human brain specimens and 1-methyl-4-phenyl-1,2,3,6-tetrahydropyridine (MPTP)-induced mouse model of PD [49]. Since then, a significant body of literature has been accumulated on the substantial roles of neuroinflammation in PD. In this regard, evidence of increased levels of inflammatory cytokines in the blood, CSF, and brain of PD patients [8] and modifications in cellular immunity such as alterations in T regulatory/T effector ratio in the blood of PD patients [50, 51] potentially confirmed the previous observations. Besides, studies on animal models of PD and neuroimaging approaches in humans provided further clues on the role of neuroinflammation in PD [52, 53].

Qin et al*.* conducted a systematic review and meta-analysis, pooling data from 25 eligible studies covering 1547 patients with PD and 1107 controls, to determine PD patients' blood levels of cytokines. They found significantly higher peripheral concentrations of IL-6, TNF-α, IL-2, IL-10, IL-1β, C-reactive protein (CRP), and regulated on activation normal T-expressed and secreted (RANTES) in patients with PD compared with the control group [54]. However, this survey neglected the effects of some essential factors, including disease duration, disease severity, and concomitant therapies’ effects on the cytokine levels of PD patients [54]. Accordingly, a prospective cohort study on 230 incident PD patients and 93 age-matched controls demonstrated significantly higher levels of TNF-α, IL1-β, IL-2, and IL-10 in PD patients [55]. This study showed that higher proinflammatory cytokine levels and lower anti-inflammatory cytokine levels were associated with more rapid progression of motor symptoms over 36 months in PD patients [55]. Therefore, this study states the hypothesis that the cytokine level might have the potential to serve as a predictive factor of PD motor progression. On the other side, the CSF levels of IL-1β and IL-6 were shown to be higher in PD patients [56]. Also, another study reported a higher concentration of IL-1β, IL-2, IL-4, TGF-α, and IL-6 in ventricular CSF of PD patients compared to controls [55]. However, the correlation between CSF and blood levels of cytokines in PD patients should be elaborated on in future studies.

**Fig. 2:**
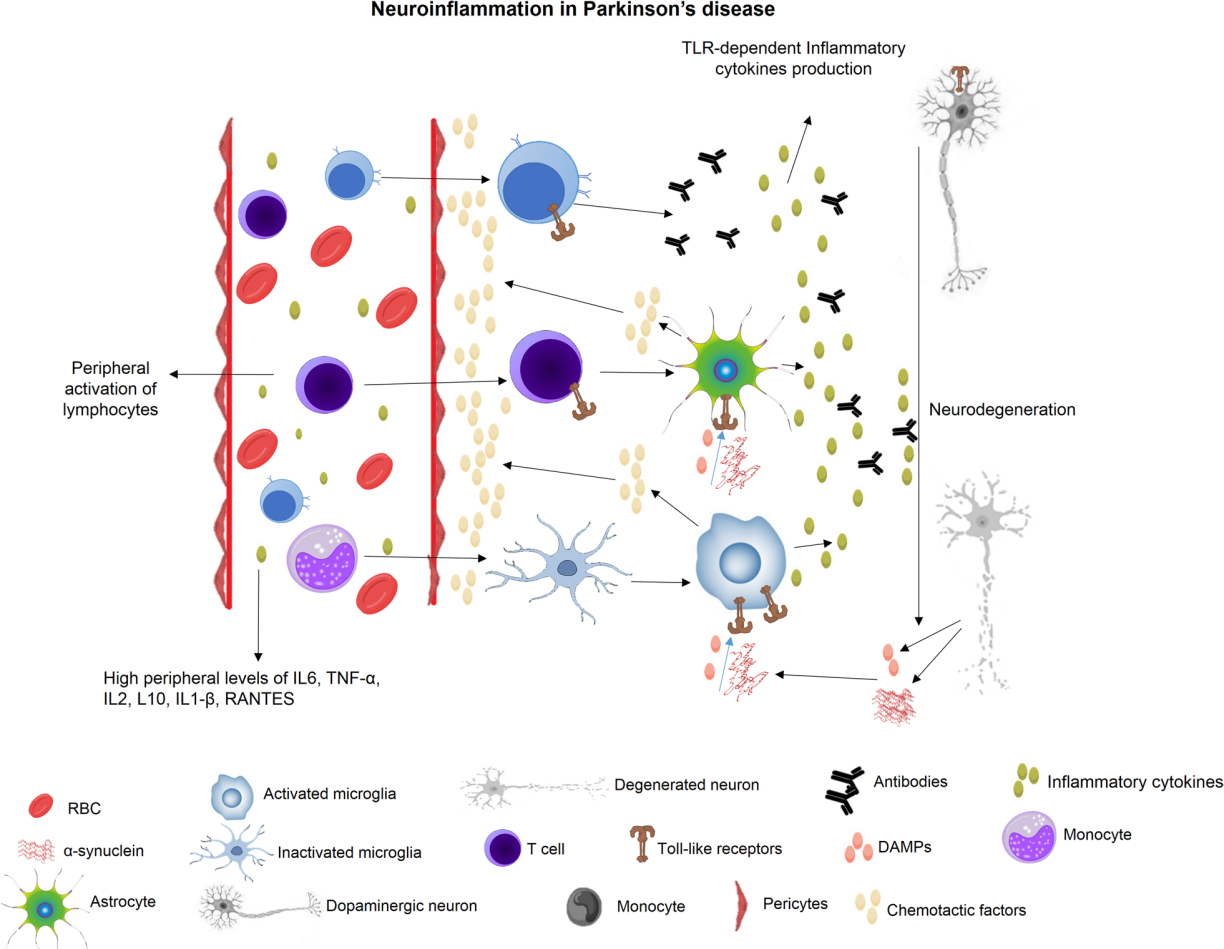
In PD patients, proinflammatory cytokines, including IL-6, TNF-α, IL-2, IL-10, IL-1β, and RANTES in the blood, CSF, and the brain tissue, are higher than in the normal population. There are also more active microglia and astrocytes in the brain of PD patients than in normal people. Inflammatory factors secreted by such cells chemotactically attract peripheral inflammatory cells, including monocytes and T and B lymphocytes, which secrete proinflammatory cytokines and antibodies and contribute to neuroinflammation. The neuroinflammation ultimately leads to degeneration of dopaminergic neurons, worsening PD symptoms. Substances released by neuronal degradation, such as DAMPs and α-synuclein, further stimulate brain-resided inflammatory cells and reinforce neurodegeneration. *TNF-α* tumor necrosis factor α, *RANTES* regulated on activation normal T-expressed and secreted, *IL* interleukin, *TLR* toll-like receptor, *DAMPs* damage-associated molecular patterns, *RBC* red blood cell

Alterations in the function of cellular immunity in PD have been reported in several studies. For example, memory T cells reacting to the α-synuclein epitopes have been detected in the serum of PD patients [57]. It has also been shown that these memory T cells can appear even before the start of clinical manifestations of the disease, and therefore it would be worthwhile to determine their efficacy as early PD biomarkers [58]. The other evidences of altered cellular immunity in PD patients were shown to be monocytes phagocytosis defection [59], dysregulation of peripheral monocytes and their hyperactivity in response to LPS [60], and the modified ratio of Teff/Treg in the serum of PD patients [51]. Moreover, a decreased concentration of naïve T cells, including CD45RA^+^ T cells, and an increased level of activated T cells like CD45RO^+^ T cells have been detected in the serum of PD patients, indicating a peripheral activation of lymphocytes [53] (Fig. 2).

Imaging studies provided further clues on the involvement of neuroinflammation in PD pathology. For example, Gerhard et al*.* used [11C] (R)-PK11195 marker, a positron emission tomography (PET) marker binding to peripheral benzodiazepine binding sites (PBBS), which is expressed mainly in mitochondria of activated microglia. They found that [11C] (R)-PK11195 was significantly more likely to bind to the basal ganglia, pons, and temporal and frontal cortical regions in patients with PD compared to the control group, suggesting the raised number of activated microglia in these regions [52]. However, by following the patients for two years, they could not find an alteration in the binding of the marker, indicating that it cannot be used as a monitoring tool for disease progression [52]. This result has been repeated by Terada et al*.* using [11C] DPA713 PET marker of microglial activation. In their small cohort, they found increased uptake of this tracer in the occipital, temporal, and parietal cortex of PD patients, which, unlike the Gerhard study, was increased in the second scan after 1 year [61]. Moreover, in 2019, a PET marker named [11C] CPPC was developed, which is a high-affinity ligand, attaching specifically to macrophage colony-stimulating factor 1 (CSF-1), the expression of which is limited to microglia. This marker demonstrated its efficacy for recognizing the microglia component of neuroinflammation in murine and non-human primate AD models. Therefore, it might be worthwhile to investigate its effectiveness in human subjects with PD in future studies [62].

Animal studies have also contributed to a better understanding of the pathophysiological and etiological roles of neuroinflammation in the disease. Animal models of PD are obtained through several methods using neurotoxin or inflammatory challenges. For obtaining neurotoxin-induced animal PD models, intracerebral injection of 6-hydroxydopamine (6OHDP) or peripheral injection of several types of complex-1 inhibitors such as MPTP, rotenone, and annonacin is most effective [53]. Moreover, polyinosinic: polycytidylic acid (poly I:C) and LPS were utilized for achieving inflammatory-induced PD animal models [63]. Also, transgenic mice overexpressing human α-synuclein genes are another useful animal model for investigating PD pathogenesis [64]. Microglial activation was the most experimental feature in the mentioned animal models of PD. For instance, microglial activation was detected following peripheral MPTP injection in mice and monkeys [65, 66]. MPTP injection chronicity also plays an essential role in the degree of microglial activation, as shown that chronic peripheral injection of MPTP caused less microglial activation than acute injection [53]. Moreover, microglial activation was also observed in human α-synuclein-overexpressing transgenic mice [67]. Therefore, the inducement of Parkinsonism through injection of neurotoxins, inflammogens [68], or manipulation of PD-associated genes could lead to microglial activation in animal studies. Several animal models have also demonstrated the association between this activation and neuronal degeneration [49, 69]. On the other side, infiltration of peripheral immune cells such as CD8^+^ or CD4^+^ T cells into the nigrostriatal system can further contribute to neuronal degeneration, as shown by a mice model of MPTP [49].

## TLRs and PD

As mentioned earlier, accumulating evidence highlighted the role of neuroinflammation in PD pathogenesis. On the other side, with a better understanding of various inflammatory effects of TLRs over the last decade, scientists have begun to think about the possible involvement of TLRs in this disease. Although the precise mechanism of TLR contribution to PD neuroinflammation is still unclear, some theories pointed to the possible role of these receptors in recognizing α-synuclein aggregates as DAMPs, which can induce proinflammatory downstream pathways, leading to neuroinflammation [70]. Such hypotheses have led to designing several studies in recent years. These studies mainly compared the expression of different TLRs and the TLR-mediated responses between PD cases and control groups using in vivo or in vitro methods. In this regard, scientists primarily work on various PD animal models like neurotoxin- or inflammatory-induced mice models of PD, microglial cells extracted from PD mice models like BV2 microglial cell line using in vitro studies [71], postmortem human brains of patients with PD, or serum of PD patients. Researchers also used different TLR agonists or antagonists in PD animal models to observe the changes in PD-related motor behaviors or compare the severity of inflammatory responses and neurodegeneration between PD cases and controls. The results of most of these studies indicated the prominent roles of TLR2, TLR4, and TLR9 in the pathogenesis of PD, with scarce evidence of the possible involvement of other TLRs, including TLR7 and TLR8 [72]. For shedding more light on this issue, the following section firstly reviewed the strengths and weaknesses of different animal models of PD; then, we reported evidence of the contribution of TLR2, TLR4, TLR9, and other TLRs to PD pathophysiology (Table 1). Afterward, we presented the various effects of different drugs on TLRs in clinical, animal, and in vitro studies (Table 2).

### Animal models of PD

Selection of a proper animal model in animal studies is crucial because the attribution of the results to humans depends highly on using appropriate animal models to imitate the human disease or condition. In this regard, along with our expanding knowledge of PD pathogenesis and manifestations, different PD animal models have been developed; each has its strengths and weaknesses. In other words, as the precise mechanism of PD is still unclear, development of a complete animal model is impossible, and all established PD animal models have disadvantages limiting their use as optimal PD models. To date, two main categories of PD animal models are applied in PD research, including neurotoxin models and genetic models.

Neurotoxin models are the most classic and most commonly used animal models of PD. The popularity of this model is due to its relatively simple way of establishment [73]. The most widely used neurotoxins for making these models are 6-OHDA and MPTP [73]. As 6-OHDA cannot penetrate BBB, it should be directly injected into the brain parenchyma. Depending on the target site of injection, including SN, striatum, or medial forebrain bundle, 6-OHDA can cause severe and quick or slow and progressive symptoms. 6-OHDA administration in the brain leads to the production of reactive oxygen species, which are thought to be responsible for the degeneration of DA neurons. Hence, this model lacks Lewy-related pathology [74]. On the other hand, MPTP can easily penetrate BBB and, therefore, it can be systematically administered. After entering the brain, MPTP is enzymatically converted to MPP + , absorbed by receptors of dopaminergic neurons. Within DA neurons, MPP + affects mitochondrial function leading to oxidative stress and degeneration of DA neurons [73, 75]. Despite other PD animal models, the MPTP model causes gradual appearance of induced symptoms, which can be considered as its strength point [73]. Although neurotoxin models are beneficial as they are easy to make, they cannot imitate any pathological features of PD; instead, they solely mimic PD symptoms. Hence these models cannot be appropriate for designing preventive treatment and clarifying the PD pathogenesis. Furthermore, they just demonstrate the late stages of PD, as the motor symptoms usually appear when severe dopaminergic degeneration has happened [74].

Genetic models are relatively new types of PD animal models established by manipulating several genes thought to be connected to PD pathogenesis. In fact, the establishment of transgenic animal models was preceded by identifying different human mutations responsible for familial cases of PD. In this regard, several genes causing mitochondrial dysregulation play their parts [76]. Moreover, specific genes, including α-synuclein, Parkin, PTEN-induced putative kinase 1 (PINK1), and DJ-1, thought to be causally linked to PD pathogenesis, were applied in developing different PD genetic models. By helping new technology, the knockout mice model of these genes could be generated, which could resemble more similarly human PD pathophysiology [77]. Moreover, different genetic models enabled us to study different genes whose expressions are changed in PD patients. Besides, genetic models of PD allow us to administer treatment before the onset of PD symptoms, having a significant role in developing preventive treatments. One of the disadvantages of genetic PD models is that many splicing events are not conserved between humans and mice. Therefore, the results of PD genetic models might not be fully applicable to humans [78]. Recently, a combined neurotoxin and genetic model has been developed, by which neurotoxins are administered to genetically manipulated animal models [79]. This model seems to be more promising and can resemble more closely human PD mechanisms. However, research on this combined model is in its infancy, and more studies are needed to examine its utility. In summary, none of the PD animal models can completely resemble human PD mechanisms, and each animal model's findings should be interpreted with caution considering its advantages and disadvantages.

### TLR2

Several studies reported an upregulation of TLR2 expression in different regions of PD postmortem human brains. For example, Drouin-Ouellet et al*.* found an increased expression of TLR2 in the caudate and putamen of PD patients [80]. In line with this, Dzamko et al*.* showed that the increased expression of TLR2 on neurons located in the anterior cingulate cortex and striatum of PD human brains is positively correlated with pathological α-synuclein accumulation and the disease staging and burden [81]. They also found that activated TLR2 promotes endogenous α-synuclein aggregation, having roles in autophagy/lysosomal pathways [81]. As TLR2 is mainly expressed on microglia, it has been suggested that the neuronal expression of this receptor might have the potential to serve as a PD-specific biomarker [70]. On the other side, TLR2 expression was shown to positively correlate with the amoeboid type of microglia in the hippocampus and SN of PD patients, highlighting its role in the microglia-mediated inflammatory responses, leading to neuroinflammation in PD [82]. Although most studies reported the roles of microglial or neuronal TLR2 in PD, emerging evidence also underpinned the significant role of astrocytes-located TLR2 in the disease-induced neuroinflammation by intensifying the inflammatory responses of these cells to the disease stimulants, including α-synuclein [83].

To investigate features of different molecular forms of α-synuclein, Gustot et al*.*, in a survey on human monocyte cell line treated with α-synuclein and anti-TLR2 antibodies, demonstrated that α-synuclein could activate TLR2 only when it folded as amyloid fibrils [84]. However, another study on human monocytes treated with anti-TLR2 before α-synuclein administration showed that both fibrillar and monomeric α-synuclein could activate TLR2 [85]. Although this discrepancy might arise from the lack of consistency in definitions of monomers, oligomers, and fibrils of α-synuclein across various studies, this issue remains to be further dissected in future clinical research.

The contribution of TLR2 to PD pathogenesis in human studies has also been confirmed by showing its higher expression on peripheral blood mononuclear cells (PBMCs) in the serum of PD patients compared with healthy controls [80, 86]. Furthermore, additional evidence of TLR2 involvement in PD pathogenesis in humans was obtained from a study indicating the correlation between TLR2 single nucleotide polymorphisms (SNPs) and the risk of PD development in a Chinese Han population [83].

In vitro and in vivo animal studies on PD also provided further evidence of the roles of TLR2, having the potential to be translated into human PD pathogenesis. In this regard, an in vitro study showed that stimulation of the murine BV2 microglial cell line with incubated recombinant human wild-type α-synuclein (SYNTR) leads to overexpression of the *TLR2* and *TLR3* genes [87]. This study also reported similar gene expression changes in primary microglia obtained from SYNTR-induced mice brain cortices [87]. Daniele et al*.* also indicated that oligomers of α-synuclein can activate microglia by engaging TLR2/TLR1 cell-membrane heterodimers, leading to translocation of NF-kB to the nucleus and proinflammatory cytokine production in BV2 mice microglia cell line [88].

A study on human α-synuclein-overexpressing transgenic mice showed that anti-TLR2 therapy reduced α-synuclein aggregation in neuronal and astroglial cells and alleviated neuroinflammation and neurodegeneration [89]. In vitro investigation in the same study uncovers the anti-TLR2 therapy efficacy through blocking neuron-to-astrocyte and neuron-to-neuron transmission of α-synuclein [89]. Another study has confirmed this result using the transgenic mice model of PD and confirmed that TLR2 activation leads to α-synuclein accumulation in neurons by inhibiting autophagy activity through the phosphatidylinositol-3-kinase mammalian target of rapamycin (AKT/mTOR) pathway [90]. In this regard, this study proposed that neuronal TLR2 regulates autophagy and *TLR2* gene ablation results in reduced neuronal α-synuclein aggregation and improved motor functions [90]. In another study, pretreatment immunization with anti-TLR2 in wild-type (WT) acute mice model of PD led to alleviation in α-synuclein-induced memory deficits [91].

Moreover, several animal studies imply that MPTP-induced mice models showed an upregulation in TLR2 [92–94]. In their survey on WT and TLR2-/- mice, Kim et al*.* demonstrated that TLR2 acted as the receptor of neuron-secreted α-synuclein, thereby mediating microglial inflammatory responses, leading to dopaminergic neuron loss in this genetic animal model of PD [90].

### TLR4

Human postmortem brain investigations by transcriptomic data analysis indicated a higher expression of TLR4 and its adaptor protein MyD88 in different brain regions of PD patients, with the most pronounced upregulation in SN and putamen [70, 95, 96]. In this regard, Kouli et al*.* found a concomitant upregulation of proinflammatory cytokine of IL-1β and TLR4 expression in the frontal cortex and SN of PD human postmortem brains [97]. In the same study, increased TLR4 expression in the amygdala was correlated with the increased CD4^+^ infiltration, higher number of activated microglia, and upregulated α-synuclein [97]. This increased TLR4 expression was also detected in peripheral immune cells of the PD patients [80, 96] as a study showed an enhanced expression of TLR4 in circulating monocytes and B cells of PD patients compared to controls [80]. Moreover, a study comparing the colonic biopsies of patients with PD and the control group detected a higher TLR4 expression in colonic samples of PD patients. This higher TLR4 expression was accompanied by a disruption in the intestinal barrier, increased microbial markers, and a higher proinflammatory gene profile in the colonic samples of PD patients [98]. Additionally, human studies found an association between SNPs of the *TLR4* gene and sporadic PD in the Chinese Han population, serving as another evidence of TLR4 involvement in PD [99].

In vivo and in vitro studies using different animal models of PD provided further evidence of the contribution of TLR4 in these models. A study showed an increased vulnerability of WT mice brain than TLR4-deficient mice brain to DA neurodegeneration following MPTP injection [100]. In line with this, an in vivo study on the MPTP mice model reported that the absence of TLR4 inhibited dopamine depletion, reduced α-synuclein-positive neurons, alleviated PD-associated neuroinflammation modifications in inflammasome pathways, and induced changes in the performance of transcription factors such as NF-κB and activator protein 1 [101]. Moreover, a study on various PD mice models using the intra-striatal injection of neurotoxins or inflammogens, including 6-OHDA, rotenone, LPS, and poly I:C reported a significant upregulation in striatal TLR4 expression at day 28 after 6-OHDA, day four after LPS, and day 14 after poly I:C injections. This study showed that TLR4 upregulation occurred earlier following injection of inflammogens like LPS, or Poly I:C, and at a later time following neurotoxin challenge with 6-OHDA. These findings suggest that the induced neuroinflammation following neurotoxin challenge might be secondary to toxin-caused neurodegeneration and the release of different inflammatory stimulant molecules such as HSP-1 and chemokines [102]. On the other side, the triggered neuroinflammation after the inflammatory challenge was suggested to be caused by the rapid development of interactions between TLR4 and administered PAMPs (LPS, or Poly I:C) [102].

However, animal studies showed some controversy concerning the roles of TLR4 in a neuroprotective or detrimental manner in PD models. For example, a study showed that α-synuclein is upregulated in the brain of TLR4 knockout mice compared with WT mice [103]. This result was repeated in a multiple system atrophy mouse model, showing that TLR4 ablation exacerbates DA neuronal loss and motor deficits [104]. Moreover, another study comparing rotenone-induced WT and TLR4 knockout mice reported that TLR4 ablation improved the SN neuronal loss and motor deficits [98]. These findings may implicate the substantial roles of TLR4 in inducing phagocytosis, which can lead to α-synuclein clearance in the acute phase of the disease. To examine this theory, Stefanova et al*.* administered anti-TLR4 therapy in BV2 mice microglia cell line. They observed that TLR4 ablation inhibited α-synuclein phagocytosis by microglia leading to α-synuclein-associated neurodegeneration and exacerbation of motor deficits [105].

In contrast, MPTP-induced mice models of PD revealed an increased TLR4 expression (70). Moreover, ablating TLR4 through silencing its genes or administering small interfering RNAs (siRNAs) in these models improved MPTP-induced motor deficits, pointing out TLR4 detrimental effects on the disease [70]. For instance, it was shown that TLR4 knockout mice are less prone to MPTP-induced toxic effects, as they showed less DA neuronal damage and fewer activated nigral microglia following the MPTP challenge [70, 72]. Moreover, Zhou et al*.* demonstrated in an in vitro study that silencing *TLR4* genes through siRNA led to a reduction in TLR4-dependent NF-kB activity and could thereby decrease microglial activation [106].

In addition, recent studies implicated the significant roles of TLR4 in recognizing α-synuclein and initiating inflammatory responses. In line with this discussion, the contribution of TLR4 to α-synuclein recognition was reported to be even more prominent than the contribution of TLR2. Hughes et al*.* revealed that oligomeric α-synuclein-induced microglial TNF-α production in WT mice was 10 to 100 times greater than in TLR4 knockout mice. They also showed that the reduction in TNF-α production in TLR2 knockout mice was not as much as in TLR4 knockout mice, suggesting the primary roles of TLR4 in α-synuclein recognition and initiation of inflammatory pathways [95]. Other studies have confirmed these results showing that TLR4-deficient mice produced lower amounts of TNF-α and ROS [13]. Moreover, another study revealed TLR4-mediated astroglial activation in mice following the exposure to purified human α-synuclein, leading to NF-kB and AP-1 pathways, Jun N-terminal kinase activation, and secretion of inflammatory cytokines and nitric oxide [107].

In line with the mentioned human study reporting higher TLR4 expression in colonic samples of PD patients, rotenone-induced TLR4 knockout mice showed reduced intestinal inflammation, intestinal dysfunction, and neurodegeneration compared with WT mice [98]. These results might point out the possible contribution of TLR4 in intestinal dysfunction and the roles of the microbiome and the gut–brain axis in disease development, which remain to be further investigated in forthcoming studies [98].

In summary, it is proposed that TLR4 has both detrimental and neuroprotective effects in PD pathogenesis, depending on the investigated time-point of the disease. In the acute phases of the disease, TLR4 contributes to α-synuclein recognition. It activates the phagocytosis response of glial cells, induces necessary inflammatory cytokines production, leading to α-synuclein clearance, and delays the disease progression. On the other side, in chronic phases of PD, excessive stimulation of TLR4-mediated proinflammatory cytokines production results in neuroinflammation, which in turn can lead to neurodegeneration, resulting in disease progression.

### TLR9

Emerging evidence also suggested a partial involvement of TLR9 in the course of PD. For instance, Ros-Bernal et al*.* detected TLR9 upregulation in the striatum of PD patients [108]. Polymorphisms in the *TLR9* gene also support the evidence of TLR9 involvement in PD. In addition, a study found that the rs352140 T allele of *TLR9* is associated with a reduced risk of PD in the Northern Iranian population [109]. In line with this result, a Chinese study found that the mentioned allele is associated with a reduced risk of PD in the Chinese Han female population [110].

On the other side, an animal study demonstrated that MPTP-intoxication in mice could lead to TLR9 upregulation [108]. In another study, TLR9 ablation in mice showed its protective effects against intoxication with MPTP [111]. Furthermore, less dopaminergic neuron death occurred in the SN of TLR9 knockout mice compared with WT mice following MPTP infusion [111]. In the same study, CpG-ODN, a TLR9 agonist, induced degeneration of dopaminergic neurons in mice lacking glucocorticoid receptors in microglia. The authors of this study proposed that glucocorticoid receptors may have some protective effects in this PD model, which should be investigated as potential therapeutic targets in future studies [111].

### Other TLRs

With respect to the evidence of the involvement of TLRs other than TLR2, 4, and 9 in PD, a human study showed that blood leukocytes' TLR7 and TLR8 responses are impaired in PD patients [72]. Moreover, an animal study using different rat models indicated a striatal upregulation of TLR3 expression following the injection of rotenone, LPS, Poly I:C, and 6-OHDA [102]. Another study showed that stimulation of murine BV2 microglial cells with α-synuclein leads to increased expression of *TLR1* and *TLR7* genes [87]. Nevertheless, research on the roles of TLRs other than TLR2, 4, 9 is in its infancy, and this subject is recommended to be dissected in detail through future studies.

**Table 1:** Evidence of the involvement of TLRs in PD through different studies

Study design	Type of evidence	Year	Most pronounced result	Conclusion	References
Comparing two genetic variants of TLR2 in the Han Chinese population	Clinical	2017	The variant allele T of the rs3804099 was higher in sporadic PD cases than rs3804100 allele	Single nucleotide polymorphism of TLR2 is associated with the development of sporadic PD in the Han Chinese population	[83]
Comparing the blood cells of PD patients and control group in response to TLR3 or TLR7,8 agonists	Clinical	2016	Patient blood cells produced lower cytokine levels after administration of TLR2 and TLR7/8 compared to the control group	Blood leukocyte TLR2 and TLR7/8 are impaired in PD, whose association with PD brain damages should be investigated in future studies	[149]
Using flow cytometry and western blot to find TLR2 and TLR4 expression in blood and brain of PD patients	Clinical	2014	Increased expression of TLR2 and TLR4 in circulating monocytes, and increased TLR4 in B cells and caudate and putamen brain regions in PD patients	TLR2 and TLR4 are modulated in the blood and brain of PD patients	[150]
Examining the expression of TLR2 in postmortem brain tissue from PD patients and matched controls	Clinical	2017	TLR2 is increased in PD brain its level correlates with the α-synuclein accumulation, the neuronal TLR2 expression (but not glial expression) was associated with PD staging	The increased expression of TLR2 on neurons might serve as a target for PD therapy	[151]
Comparing the phenotype and TLR2 expression between PD patients and incidental Lewy body disease (iLBD) cases and control group	Clinical	2014	Pronounced increase of microglial TLR2 expression in iLBD cases (but not PD cases) compared to control, Increase in amoeboid microglia in PD cases	TLR2 may play a significant role in microglia-mediated responses in PD	[152]
In vitro study, comparing the response of murine TLR4-knockdown microglia and wild-type microglia to Paraquat	Clinical	2020	Paraquat-induced production of inflammatory cytokines was significantly reduced in TLR4-knockdown microglia	TLR4 contributes to neuroinflammation in the Paraquat-induced model of neurodegeneration	[153]
Comparing postmortem brains of PD dementia (PDD) and PD with no dementia (PDND) patients and control group	Clinical	2020	Upregulation of TLR4 in the substantia nigra, frontal cortex, and amygdala in both PDD and PDND patients compared to control group	TLR4 contributes to neuroinflammation in PD	[97]
Comparing the colonic biopsy samples of PD patients and control group	Clinical	2019	Higher expression of the endotoxin-specific ligand TLR4, CD3 + T cells, and cytokine expression, and dysbiosis in colonic samples of PD patients	TLR4 contributes to neuroinflammation and intestine inflammation in PD	[98]
Using flow cytometry and western blot to find TLR2 and TLR4 expression in blood and brain of PD patients	Clinical	2014	Increased expression of TLR2 and TLR4 in circulating monocytes, and increased TLR4 in B cells and caudate and putamen brain regions in PD patients	TLR2 and TLR4 are modulated in the blood and brain of PD patients	[150]
Comparing single nucleotide polymorphisms of TLR9 between PD patients and control group	Clinical	2020	The DNA analysis of samples showed that rs352140 T allele of TLR9 was associated with reduced risk of PD	TLR9 SNPs are associated with PD risk	[154]
Comparing the WT and TLR4-deficient MPTP-induced mice brain regions by Fourier Transform Infrared	Animal/in vitro	2017	WT mice were more prone to dopaminergic neuron degeneration following MPTP	TLR4 play roles in biochemical changes relating to neurodegeneration in MPTP-induced animal model of PD	[100]
In vivo model of PD using MPTP mice	Animal/in vitro	2019	The absence of TLR4 prevented inflammation, cytokine production, dopamine depletion, modulated inflammasome pathway, and reduced astrogliosis, and α-synuclein-positive neurons	TLR4 may be an attractive therapeutic target for reversing PD-like manifestations in PD animal model	[155]
Male rats were given intra-striatal injections of 6-hydroxydopamine, rotenone, LPS, or Poly I:C, and the expression of TLR3 and TLR4 were examined	Animal/in vitro	2017	Prominent changes in TLR3 and TLR4 expression in the inflamed striatum of all rats	TLR3 and TLR4 play significant roles in inducing PD-like symptoms in 6OHDP-induced animal model of PD	[102]
Comparing the behavior and biochemistry of striatal and SN brain regions of MPTP-induced wild-type mice and MPTP-induced TLR4-deficient mice	Animal/in vitro	2019	TLR4 deficiency significantly improved MPTP-induced motor deficits, attenuated α-synuclein reduction, and improved neuroinflammation	TLR4 contributes significantly to PD-like symptoms in MPTP-induced animal model of PD	[156]
Comparing α-synuclein-treated TLR2 knockout mice and WT mice microglia	Animal/in vitro	2013	Extracellular oligomeric α-synuclein released from neuron cells serve as a ligand for TLR2 and initiate an inflammatory response	TLR2 and oligomeric α-synuclein both might have the potential to serve as novel therapeutic targets in PD	[157]
Comparing TLR2 knockout and WT mice	Animal/in vitro	2016	Neuron-derived α-synuclein activates TLR2 and leads to neuroinflammation-induced neurodegeneration	TLR2 is an essential molecule mediating non-cell-autonomous neurotoxic effects of α-synuclein in the genetic animal model of PD	[158]
Comparing A53T + TLR2 + / + and A53T + TLR2 knockout mice	Animal/in vitro	2016	Inactivating TLR2 led to phagocytosis activation and decreased α-synuclein aggregation Moreover, activation of TLR2 led to reduced phagocytosis activity by regulating AKT/mTOR	TLR2 plays a significant role in the phagocytosis activities of microglia	[90]

*TLR* toll-like receptor, *WT mice* wild-type mice, *PD* Parkinson’s disease, *SNP* single nucleotide polymorphism, *MPTP* 1-methyl-4-phenyl-1,2,3,6-tetrahydropyridine, *SN* substantia nigra, *LPS* lipopolysaccharide, *Poly I:C* polyinosinic:polycytidylic acid, *PDD* PD with dementia, *PDND* PD with no dementia, *iLBD* incidental Lewy body disease

### Substances with potential effects on TLRs and neuroinflammation in PD

#### Cordycepin

Cordycepin, or *3*'-*deoxyadenosine*, is a main bioactive constituent of a fungus species named *Cordyceps militaris*, extracted and used in traditional Chinese medicine [112]. The molecular structure of cordycepin is 9-(3-deoxy-β-d-ribofuranosyl) adenine, and its effects on various diseases have been extensively studied since its isolation from the *C. militaris* in 1951 [112, 113]. In this regard, cordycepin can exert a wide range of pharmacological effects, from which the anti-inflammatory, anti-cancer, neuroprotective, and anti-oxidant properties were its most observed features [112, 114]. For instance, cordycepin has shown its effects in upregulating IL-10 in human blood mononuclear cells, thereby suppressing the secretion of inflammatory cytokines [115]. Cordycepin was administered in different studies using either oral or intravenous routes [116, 117]. In this regard, the bioavailability of orally administered cordycepin can be significantly improved using nanoencapsulation of the drug combined with gastric acid inhibition strategies [118]. Cordycepin can reportedly efficiently protect BBB integrity by reducing local neuroinflammation, reorganizing tight junctions, and prohibiting the activity of NADPH oxidase (NOX) [119]. Although not completely clear, these protective effects on BBB suggest that Cordycepin probably has the ability to penetrate BBB [120]. Cheng et al*.* investigated Cordycepin's effects on PD rat models by administering Cordycepin one hour after the first MPTP injection to rats. Subsequently, they assessed motor function tests and other experiments after 15 days of MPTP injection. They demonstrated that cordycepin could alleviate MPTP-induced changes, including motor disorders, DA neuron loss, and activation of TLR/NF-kB. They also illustrated that cordycepin diminished the cytotoxic impacts of LPS on PC12 cell lines by microglia [113].

#### SA00025

Nuclear receptor-related 1 (Nurr-1) is a protein controlling the specific genes required for dopamine regulation in dopaminergic neurons in SNpc [121]. Eliminating Nurr-1 has led to dopaminergic phenotype loss in both in vitro and in vivo studies [122, 123]. Hence, Nurr-1 plays an essential role in the differentiation of dopaminergic neurons in embryonic phases and conservation of dopaminergic phenotype during the lifetime, suggesting its probable functions in PD. Additionally, Nurr-1 can reportedly mitigate inflammation, which is another risk factor for PD [124]. Therefore, increasing Nurr-1 or inducing it might exert beneficial effects on PD. SA00025 is a novel Nurr-1 agonist with molecular structure of 2-{3-[2-(4-chlorophenyl)imidazo[1,2-a]-pyridin-6-yl]phenyl}propan-2-ol, whose effectiveness was tested in an animal model of PD [124]. Smith et al*.* administered a daily 30 mg/kg dose of SA00025 by oral route to a 6-OHDA-induced model of rats. After seven consecutive days, the rats were killed. The pharmacokinetic and postmortem brain analysis showed that SA00025 entered the brain 1–48 h after the last oral administration on day 7 and induced transcriptional upregulation of Nurr-1 protein and modulated dopaminergic-related gens. This finding might suggest that SA00025 can penetrate the BBB. Furthermore, daily gavage administration of SA00025 for 32 days led to dopaminergic neuroprotective effects, induction of resting-state of microglia, and reduction of IBA-1 staining intensity of microglia, GFAP staining intensity of astrocytes, and IL-6 levels [124].

#### Peroxiredoxin 6

Peroxiredoxins (PRX) are highly conserved ubiquitous family enzymes with catalytic abilities. PRDX6 is the only member of this family capable of reducing phospholipid hydroperoxides and short-chain hydroperoxides [125]. Yeo et al*.* compared neural stem cells extracted from E15 days wild-type and PRDX6-overexpressing mice. They demonstrated that neurogenesis and expression of protein biomarkers are impaired in neural stem cells isolated from PRDX6 mice. They also showed that WD repeat and FYVE domain-containing protein 1 (WDFY1) expression was dramatically reduced in PRDX6 mice through a TLR4-dependent mechanism. In this regard, they showed the potential utility of introducing the plasmid of WDFY1 to neural stem cells of PRDX6 mice for improving neurogenesis and reversing TLR4 [126]. Collectively, this study showed that PRDX6 inhibitors might play beneficial roles in the future way of PD treatment.

#### Dihydrotestosterone

Dihydrotestosterone (DHT) is a 5α-reduced derivative of testosterone with potent androgenic activities, converted from testosterone in target tissues [127]. DHT is an injectable drug that can penetrate the BBB and affect the CNS as it has a lipophilic molecular structure [127, 128]. Yang et al*.* demonstrated the effects of DHT on neuroinflammation, impaired behavior, and neuronal damage in an LPS-induced mice model. They reported that DHT could reduce proinflammatory cytokine production in BV-2 cells and primary microglia by inhibiting TLR4-dependent NF-κB and MAPK p38 pathways, protecting neurons. Moreover, behavioral tests showed that DHT could ameliorate learning and spatial disability, motor incoordination, and locomotor abilities in LPS-induced mice models [129].

#### Hesperetin

Hesperetin is a derivative of flavanone named trihydroxyflavanone, which is found mainly in citrus fruits like oranges [130]. Hesperetin has been reported to have the ability to cross the BBB and reach the CNS [131]. Muhammad et al*.* investigated the effects of hesperetin on an LPS-induced mice model of neuroinflammation. They intraperitoneally administered seven doses of LPS in 14 days, and they orally gavaged hesperetin daily for 5 weeks starting 3 weeks before LPS injection. They observed that hesperetin treatment reduced proinflammatory cytokine secretion by reducing TLR4-dependent Iba1-GFAP expression. Furthermore, hesperetin improved synaptic integrity, cognition, and memory. These findings pointed out that hesperetin can exert neuroprotective effects using the TLR4/NF-κB signaling pathway [132].

#### Icariside II (ICS II)

Icariside II is an active flavonoid component derived from a Chinese herbal medicine named *Epimedium brevicornum* *Maxim*. This substance has been reported to exert neuroprotective effects in AD models. Moreover, it has been demonstrated that ICS II can cross the BBB [131]. ICS II has been administered orally in most animal studies [133]. Zhou et al*.* investigated the effects of ICS II on an LPS-induced rat model. They administered ICS II prophylactically seven days before intracerebroventricular injection of LPS. The findings demonstrated that pretreatment with ICS II could inhibit microglia and astrocyte activation, suppress proinflammatory cytokine production and reduce TLR4, MyD88, and TRAF expressions. Hence, ICS II could reportedly reverse LPS-induced neuroinflammation in rats, suggesting its potential therapeutic effects on neuroinflammation [134].

#### Monophosphoryl lipid A

Monophosphoryl lipid A (MPL), an LPS-derived substance, is a detoxified fraction of endotoxin lipid A, lacking a saccharide and phosphate group [134]. It can be obtained from bacteria like *Salmonella Minnesota* or by chemical synthesis from Lipid A obtained from *Escherichia coli* [135]*. *MPL is a potent, selective agonist of TLR4 and an inducer of phagocytosis without causing a severe inflammatory response compared to LPS [136]. MPL is also used in several vaccines [136]. This drug is administered via injection, and similar to LPS, it probably cannot cross the BBB [137]. Venezia et al*.* investigated the chronic systemic administration of MPLA in transgenic mice with proteolipid promoter-induced overexpressing α-synuclein (PLP-α-syn mice) model of multiple system atrophy. They concluded that this chronic treatment with MPLA led to motor improvement, higher-α-synuclein uptake by microglia, protection of nigral striatal and dopaminergic neurons, and decreased α-synuclein cytoplasmic inclusions without causing systemic inflammation [136]. These results might warrant the worth of further investigations of this drug in PD human patients.

#### Vinpocetine

Vinpocetine is a semisynthetic derivative of Vinca alkaloid vincamine, an alkaloid extracted from the periwinkle plant [138]. This substance can be administered either orally or intravenously and has the ability to penetrate the BBB and enter the CNS [139]. It has been known for its improving effects on memory, cerebral metabolism, and inducing vasodilation [140]. In a double-blind placebo-controlled trial, 89 patients with late-onset PD and 42 controls were recruited. PD patients were randomly allocated to traditional or vinpocetine PD treatment groups. However, in both groups, levodopa was administered from the first day to the last day of the study (the 14th day). Vinpocetine was administered daily to the vinpocetine treatment group. The researchers reported that vinpocetine administration could decrease mRNA levels of TLR2 and TLR4 and their downstream signaling molecules like MyD88 and NF-kB in peripheral blood monocytes. On the other side, it induced TLR3 and its downstream signaling molecules like TRIF-β and IRF-3 and serum anti-inflammatory cytokine concentrations. Additionally, vinpocetine reduced serum levels of proinflammatory cytokines. These findings may suggest that vinpocetine has beneficial anti-inflammatory effects and can improve cognitive impairment in PD patients [140]. However, it is not wholly evident whether this drug's anti-inflammatory effects or other induced mechanisms led to improved cognitive function, remaining to be elucidated in future studies.

#### CU-CPT22 and candesartan cilexetil

CU-CPT22 is a benzotropolone analog with potent inhibitory effects on TLR1/2, firstly developed by Cheng et al*.* in 2013 [141]. It has not yet been elucidated whether this molecule can cross the BBB or not. Daniele et al*.* demonstrated that inhibiting TLR1 and TLR2 with CU-CPT22 could reduce proinflammatory cytokine production in cultured BV2 microglia of mice [88]. On the other side, candesartan cilexetil is an approved angiotensin II receptor blocker used for treating hypertension. This drug poses inhibitory effects on TLRs expression, especially TLR2 and TLR4. Candesartan cilexetil is administered using the oral route and can cross the BBB. Candesartan cilexetil reversed the proinflammatory phenotype of primary mice microglia induced by oligomeric α-synuclein in Daniele’s study [88]. Moreover, candesartan cilexetil showed inhibitory effects on TLR2 and TLR4 expression and proinflammatory cytokine secretion like TNF-α, IL-6, and IL-1β, both in primary human monocyte and mice models of PD [142]. These promising effects suggest examining the translatability of candesartan effects to PD humans as an example of a drug-repurposing strategy in future clinical studies.

#### SIlibinin (a prothrombin kringle-2 inhibitor)

Prothrombin kringle-2 (PKr-2) is one of the domains of prothrombin that is cleaved and generated by active thrombin. Although PKr-2 does not directly exert a toxic effect on neurons, it could induce DA neuronal death in the SN of rats by TLR4-mediated activation of microglia [143]. In this regard, the levels of PKr-2 and TLR4 significantly increased in the SN of PD patients than in controls. These findings suggest that inhibition of PKr-2 might protect DA neurons from degeneration by suppressing TLR4-mediated microglial activation. In this regard, treatment with silibinin, a polyphenolic flavonoid mainly found in milk thistle, could reportedly reverse the pathological effects of PKr-2 in SN of rat brains, leading to DA neurons’ protection. Silibinin can cross the BBB and can be administered orally [144].

#### Asiatic acid

Asiatic acid is a pentacyclic triterpene found in several plant foods like Centella and olive [145]. AA was reported to have beneficiary effects on wound healing, cancer, and neurodegenerative diseases [146]. This substance can penetrate the BBB and enter the CNS [147]. AA has shown acceptable pharmacokinetics following oral or intraperitoneal administration [146]. In a study on the MPTP mice model of PD, the oral administration of AA reduced MPTP-induced apoptotic, inflammatory, and oxidative effects and also attenuated α-synuclein, TLR2, TLR4, and NF-kB expression, increased striatal levels of BDNF, dopamine, and neurotrophic factors in a dose-dependent manner [92]. However, this question has remained whether these effects can be translated to human PD.

#### Fecal microbial transplantation (FMT)

Evidence supports a connection between the gut microbiota composition and the onset or progression of PD [148]. In this regard, Sun et al*.* demonstrated that the microbial composition of the PD model of mice differs significantly from normal mice. However, fecal microbiota transplantation to the PD mice model showed significant improvements in physical impairment and elevation in striatal DA and 5-Ht components of the PD mice model. They also observed that FMT could reverse the microglia and astrocyte activation in SN and decreased TLR4 and TNF-αexpression in the gut and brain of the PD mice model [148].

**Table 2:** Substance affecting TLRs and neuroinflammation in PD

Name of substance	Type of evidence	Sample	Effect of therapy	Year	References
Vinpocetine	Clinical	A cohort of 89 Parkinson’s disease patients and 42 healthy controls	Administration of vinpocetine reduced mRNA levels of TLR2/4 and protein levels of MyD88 and NF-kB; and it reduced the levels of serum inflammatory cytokines	2019	[140]
Cordycepin	Animal/in vitro	MPTP-induced murine model and LPS-induced cell model of PD	Cordycepin improved PD symptoms by inhibiting TLR/NF-kB signaling pathway in vivo and in vitro	2019	[113]
SA00025 (a nuclear receptor-related 1 agonist)	Animal/in vitro	Rats administered by a TLR3 agonist (poly I:C) and 6-OHDA	It caused neuroprotective and anti-inflammatory effects in animal model of PD	2015	[159]
Peroxiredoxin 6	Animal/in vitro	PRDX6-overexpressing transgenic (Tg) mice and wild-type mice	PRDX6 inhibited the neurogenesis by TLR4-dependent pathway contributing to symptoms in this genetic model of PD	2019	[126]
Fecal microbial transplantation (FMT)	Animal/in vitro	PD mice	FMT reduced expression of TLR4/TNF-α signaling pathway components in gut and brain and suppressed neuroinflammation	2018	[160]
Dihydrotestosterone (DHT)	Animal/in vitro	LPS-induced mice model	DHT induced anti-neuroinflammatory and neuroprotective effects, inhibiting inflammatory cytokine production through TLR4	2020	[129]
Hesperetin	Animal/in vitro	LPS-induced BV2 cells of mice	Hesperetin treatment alleviated proinflammatory cytokine production by ameliorating TLR4-mediated ionized calcium-binding adapter molecule 1/glial fibrillary acidic protein (Iba-1/GFAP) expression	2019	[132]
Icariside II (ICS II)	Animal/in vitro	LPS-infused rats	ICS could attenuate LPS-induced neuroinflammation by inhibiting TLR4/MyD88/NF-kB pathway in rats	2019	[134]
Monophosphoryl lipid A (a TLR4 agonist)	Animal/in vitro	Transgenic mice overexpressing α-synuclein (proteolipid protein promoter-α-syn mouse model)	Chronic systemic MPLA treatment led to increased uptake of α-synuclein by microglial cells, pronounced motor improvement, and rescue of DA neurons	2017	[161]
CU-CPT22 (candesartan cilexetil)	Animal/in vitro	BV2 mice microglial cells	CU-CPT22 reduced the nuclear translocation of NF-κB and secretion of TNF-α from cultured primary mouse microglia Candesartan cilexetil reversed the proinflammatory phenotype of primary mice microglia induced by α-synuclein	2015	[88]
Prothrombin kringle-2 (pKr-2)	Animal/in vitro	Rat and mouse brain injected with pKr-2	Microglial TLR4 was upregulated in the rat SN and in cultures of the BV2 microglial cell line after PKr-2 treatment	2015	[162]
Asiatic acid (AA)	Animal/in vitro	MPTP mouse model of PD	AA treatments reduced striatal expression of α-synuclein and TLR4, increased striatal levels of dopamine, brain-derived nerve growth factor, and glial cell line-derived neurotrophic factor	2016	[92]

*TLR* toll-like receptor, *MPTP* 1-methyl-4-phenyl-1,2,3,6-tetrahydropyridine, *PD* Parkinson’s disease, *pKr-2* prothrombin kringle-2, *NF-κB* nuclear factor kappa-light-chain-enhancer of activated B cells, *mRNA* messenger RNA, MPLA: monophosphoryl lipid A, DA: dopaminergic, TNF-α: tumor necrosis factor-α, 6-OHDA: 6-hydroxydopamine, poly I:C: polyinosinic:polycytidylic acid.

## Conclusion

Neuroinflammation is one of the key contributors to PD pathogenesis, resulting in dopaminergic neurodegeneration and progression of the disease. As shown through several studies, evidence of the role of neuroinflammation in PD patients are indicative of higher levels of proinflammatory cytokines in serum and cerebrospinal fluid, presence of activated microglia in different CNS areas such as substantia nigra, infiltration of CD4^+^ and CD8^+^ T cells in affected brain regions, and modified performance of the cellular immunity for example malfunction in monocytes phagocytosis.

Postmortem studies on human brains and different animal PD models indicated an upregulation in TLRs expression in different brain regions such as the striatum, putamen, and caudate. This increased expression was also observed in peripheral blood monocytes of the affected individuals. Moreover, different SNPs in some *TLR* genes demonstrated an association with susceptibility to PD. Most studies indicated that the increased TLR expression is associated with neuroinflammation and neural loss in PD cases. In this regard, TLR2, TLR4, and TLR9 play a more significant role compared to other contributors. The increased expression of TLR2 on neurons located in the anterior cingulate cortex and striatum of PD human brains was also shown to be correlated with the disease staging and burden. TLR2 and TLR4 are critical receptors of α-synuclein, performing crucial roles in its recognition and triggering inflammatory responses. Furthermore, TLR4 is necessary for the proper phagocytosis function of monocytes, resulting in α-synuclein clearance in the early stages of the disease.

However, despite extensive research on PD and its treatments, no effective curative therapy has yet been developed. This therapeutic failure might be due to the incomplete knowledge of the disease pathogenesis. Recently, the discovery of the involvement of TLR-mediated neuroinflammation in disease pathogenesis has attracted much attention in designing novel therapeutic approaches. Although the essential roles of TLR in PD onset and progression have been illustrated in several clinical and preclinical studies, some knowledge gaps still remain to be addressed regarding the precise mechanisms of TLRs’ contribution to PD. For instance, the molecular structure of all pathological molecules responsible for initiating TLRs activation in PD patients is still not entirely discovered. Moreover, the roles of TLRs expressed on CNS-residing cells other than microglia, including astroglial and neuronal TLRs, attracted inadequate attention, necessitating to be elucidated in future studies. In this regard, more research using combined genetic and neurotoxin animal models of PD might be helpful to elucidate the roles of TLRs in disease pathogenesis more accurately.

Although the exact molecular mechanisms of TLR contribution to PD and neuroinflammation are still to be elucidated, the information presented in this review underlines the importance of designing approved drugs targeting TLRs and their downstream signaling pathways as potential therapeutic approaches in future way of PD research. In this context, the future research path on PD should be devoted to designing more randomized clinical control trials examining the efficacy of TLRs-based neuroinflammation modulating substances that showed promising results in animal and in vitro studies. However, before this point is reached, establishing validated animal models of PD, based on our expanding knowledge of the disease pathogenesis, which more closely imitates the molecular mechanisms of PD in humans, would help. Moreover, the pharmacokinetic features and the ability to penetrate BBB are of undoubted importance in designing effective drugs targeting TLRs in PD. In this regard, nanoencapsulation of developed drugs for their effective delivery to their target sites could play essential roles. Hence, there is a substantial need to examine the translatability of the effects of TLRs-affecting compounds in human clinical research.

To draw a conclusion upon the discussion, neuroinflammation and activation of TLRs function as a double-edged sword, exerting both protective and detrimental effects on PD pathogenesis. In the early time points of the disease, they might lead to α-synuclein clearance, which delays the disease progression. However, in later time points, excessive induction of TLR-mediated neuroinflammation results in neurodegeneration, worsening the disease prognosis. In line with this, different factors responsible for the dual effects of TLRs on the disease prognosis should be more thoroughly explored in future studies, with the hope of establishing novel therapeutic strategies for turning detrimental responses of TLRs into beneficial ones.

## Data Availability

Not applicable.

## Abbreviations

PD: Parkinson's disease
TNF-α: Tumor necrosis factor-α
RANTES: Regulated on activation normal T-expressed and secreted
IL: Interleukin
TLR: Toll-like receptor
DAMPs: Damage-associated molecular patterns
RBC: Red blood cell
LPS: Lipopolysaccharide
CD: Cluster of differentiation
MD2: Myeloid differentiation factor 2
MyD88: Myeloid differentiation primary response 88
IRAK4: Interleukin-1 receptor-associated kinase 4
TRAF6: TNF (tumor necrosis factor) receptor-associated factor 6
TAK1: Transforming growth factor β-activated kinase 1
MKK: Mitogen-activated protein kinase kinase
JNK: Jun N-terminal Kinase
IRF: Interferon regulatory factor
MPL: Monophosphoryl lipid A
p38MAPK: P38 mitogen-activated protein kinase
AP1: Activating protein-1
NF-kB: Nuclear factor kappa-light-chain-enhancer of activated B cells
TRIF: TIR-domain-containing adaptor-inducing interferon-β
TIR: Toll/interleukin-1 receptor
TANK: TRAF family member-associated NF-kappa-B activator
WDFY1: WD repeat and FYVE domain-containing protein 1
LBP: Lipopolysaccharide binding protein
dsRNA: Double-stranded RNA
ssRNA: Single-stranded RNA
LRR: Leucine-rich repeats
PRRs: Pattern recognition receptors
PAMPs: Pathogen-associated molecular patterns
DAMPs: Damage-associated molecular patterns
SNc: Substantia nigra pars compacta
DD: Death domain
LTA: Lipoteichoic acid
MAPK: Mitogen-activated protein kinase
BBB: Blood–brain barrier
FADD: Fas-associated protein with death domain
TOLLIP: Toll interacting protein
SHIP: SH2-containing inositol-5′-phosphatase
CIAPs: Cellular inhibitor of apoptosis proteins
PBMC: Peripheral blood mononuclear cell
Aβ: Amyloid beta
mTOR: Mammalian target of rapamycin
PGN: Peptidoglycan
MPTP: 1-Methyl-4-phenyl-1,2,3,6-tetrahydropyridine
PET: Positron emission tomography
PBBS: Peripheral benzodiazepine binding sites
TBK1: TANK-binding kinase 1
